# The Effect of Fatigue on Lower Limb Joint Stiffness at Different Walking Speeds

**DOI:** 10.3390/diagnostics12061470

**Published:** 2022-06-15

**Authors:** Enze Shao, Zhenghui Lu, Xuanzhen Cen, Zhiyi Zheng, Dong Sun, Yaodong Gu

**Affiliations:** 1 Faculty of Sports Science, Ningbo University, Ningbo 315211, China; shaoenze@hotmail.com (E.S.); luzhenghui_nbu@foxmail.com (Z.L.); cenxuanzhen@outlook.com (X.C.); sundong@nbu.edu.cn (D.S.); 2 Doctoral School on Safety and Security Sciences, Obuda University, 1034 Budapest, Hungary; 3 Faculty of Engineering, University of Szeged, 6720 Szeged, Hungary; 4 ANTA Sports Science Laboratory, ANTA (China) Co., Ltd., Xiamen 361008, China

**Keywords:** joint stiffness, walking fatigue, lower limb joints

## Abstract

The aim of this study was to assess the stiffness of each lower limb joint in healthy persons walking at varying speeds when fatigued. The study included 24 subjects (all male; age: 28.16 ± 7.10 years; height: 1.75 ± 0.04 m; weight: 70.62 ± 4.70 kg). A Vicon three-dimensional analysis system and a force plate were used to collect lower extremity kinematic and kinetic data from the participants before and after walking training under various walking situations. Least-squares linear regression equations were utilized to evaluate joint stiffness during single-leg support. Three velocities significantly affected the stiffness of the knee and hip joint (*p* < 0.001), with a positive correlation. However, ankle joint stiffness was significantly lower only at maximum speed (*p* < 0.001). Hip stiffness was significantly higher after walking training than that before training (*p* < 0.001). In contrast, knee stiffness after training was significantly lower than pre-training stiffness in the same walking condition (*p* < 0.001). Ankle stiffness differed only at maximum speed, and it was significantly higher than pre-training stiffness (*p* < 0.001). Walking fatigue appeared to change the mechanical properties of the joint. Remarkably, at the maximum walking velocity in exhaustion, when the load on the hip joint was significantly increased, the knee joint’s stiffness decreased, possibly leading to joint instability that results in exercise injury.

## 1. Introduction

Walking is one of the most popular sports and is performed by a wide range of individuals [1]. It requires little skill, has a low risk of injury, and is considered a natural, widely accepted, low-cost, and environmentally undemanding sport [2]. Murtagh et al. published research that demonstrated [3] that walking increases cardiorespiratory fitness and blood pressure management in trained persons while decreasing waist circumference, body weight, body fat percentage, and body mass index. Therefore, walking training is a practical, physical activity approach for individuals who are sedentary or overweight as a result of work stress, are at risk of cardiovascular disease, and are unable to exercise vigorously [4]. Of course, all exercise requires a specific amount of exercise load to benefit the body. For most individuals, adjusting the walking pace to a moderate speed that suits the individual allows for a more acceptable personal workout load that will efficiently excite the body and provide favorable benefits [5,6].

Studies have demonstrated a dosage connection between physical activity and health benefits [7]. Walking for 30 min at a relative intensity that controls the body’s heart rate reserve (70–85% of the heart rate reserve) has a considerable favorable influence on human function improvement. Walking has become a tactic to increase physical exercise [8]. However, it is worth noting that, during prolonged low-intensity exercise, walkers may experience impaired muscle activation that affects exercise performance while producing a psychobiological state characterized by feelings of fatigue and a lack of motivation [9,10,11]. Further investigation of lower extremity motor function following walking training is thus required. Lower extremity biomechanical monitoring studies have been conducted [12,13,14]. Lower limb stiffness is a reliable reference measure for evaluating the dynamic properties of the entire lower limb during walking or running, and it is a crucial parameter for describing human motor performance.

Mechanical stiffness in the human body is divided into quasi-stiffness and joint stiffness. Quasi-stiffness is the body’s ability to prevent external displacement without considering the distribution of displacement over time [15]. Common types of quasi-stiffness for motion analysis in humans and animals are leg and vertical stiffness, and the parameters above can be used to describe the mechanical properties of the subject’s lower limbs [16]. Joint stiffness (k_joint_) is the mechanical force that resists displacement inside the corresponding joints of the lower limbs (e.g., the hip, knee, and ankle), which is determined by the physiological structure of the corresponding joints, the mode of motion, and the mechanical characteristics of the joints [17]. Mechanical stiffness has been widely employed in biomechanics research and is often analyzed during cyclic (e.g., running and walking) and single (e.g., long jump and vertical leap) motions [18,19,20]. Athletes’ lower limb injuries may occur if mechanical stiffness is too low or too high [21]. Stiffness that is too low may produce excessive joint mobility and injury to the related soft tissue [22,23,24]. Furthermore, excessive stiffness may raise the risk of lower extremity bone damage [25]. Vertical stiffness is widely used to explain the stiffness of the lower limb [26]. However, further movement strategy research discovered that, while the stiffness of individual lower limb joints may vary, the vertical stiffness remains constant [27]. Simultaneously, measuring the stiffness of each lower limb joint is a method of indirectly determining if the lower limb is being overused [28]. It should be emphasized that joint stiffness is connected to muscle stiffness and can thus be active or passive [29]. In past findings, passive stiffness was characterized as a feature of joint structures that did not involve muscle activation [20,29]. Dynamic stiffness is the change in muscle activity that occurs during the segment-to-segment displacement time under consideration [29,30].

Previous gait research has found variations in ankle [31,32], knee [33,34], and hip stiffness [35] during normal walking. Jin and Hahn [33] underlined the importance of investigating the relationship between joint stiffness and leg stiffness during different locomotion tasks and speeds. They indicated that it may be beneficial to further investigate the relationship between joint stiffness and leg stiffness during different locomotion tasks and speeds. In addition, gait characteristics after walking fatigue were investigated, facilitating the evaluation of postoperative gait and anti-fatigue function in patients [36]. Most research on lower extremity joint stiffness has centered on running and other sports [37,38,39]. However, the changes in lower limb joint stiffness after walking fatigue and the influence of walking speed on joint stiffness in exhaustion remain unknown. Simultaneously, in earlier gait speed studies [33,40,41], the control of walking speed was between 1 and 2 m/s. To satisfy the maximum walking speed requirement, some studies have simultaneously examined the three joints of the lower limb to determine whether their dynamic stiffness exhibits a distinct performance (a walking speed greater than 2 m/s).

This study aimed to compare the dynamic stiffness of each lower limb joint before and after walking fatigue when walking at varying speeds in healthy persons. The influence of velocity and fatigue on lower extremity dynamic joint stiffness during single-leg support was investigated. The study reveals more information about the adaption patterns of lower limb biomechanics while walking by observing the variations in dynamic joint stiffness before and after walking fatigue.

## 2. Materials and Methods

### 2.1. Participants

This study included 24 individuals (all male; age: 28.16 ± 7.10 years; height: 1.75 ± 0.04 m; weight: 70.62 ± 4.70 kg). All participants in the research had no scientific walking training experience and performed walking tests below the level of higher-level athletes. Before data collection, subjects were familiar with the walking experiment procedure and the Borg Scale RPE 6-20 testing requirements [42]. Of all the participants, there was no lower extremity or back musculoskeletal discomfort or injury lasting more than one week at any point six months before participation in the trial, no history of lower extremity or back surgery, and no current usage of foot orthotics. Ningbo University’s review committee approved this work (No. RAGH202108293306). Before participating, all participants completed a written informed consent form.

### 2.2. Walking Training

Before data collection, participants were instructed on how to use the Borg Scale RPE 6-20 scale, which was used in conjunction with a heart rate monitor (Polar RS100, Polar Electro Oy, Woodbury, NY, USA) to record subjective weariness and heart rate variations following walking exercise. A certified physical trainer guided participants through a 10-min warm-up session. Following the warm-up, participants walked on a motorized treadmill (h/p/cosmos sports and medical GmbH, Nussdorf-Traunstein, Germany), with the treadmill controlling the walking speed. The walking training speed increased as the walking kilometers increased, and for every 400 completed steps, the walking speed increased by 1 km/h. Meanwhile, after every 400 m of walking training, the observer recorded the participant’s heart rate and the Borg Scale RPE 6-20 scale. Walking fatigue was identified [43], and walking training was discontinued when all of the following requirements were met: (1) the participant’s heart rate surpassed 90% of the maximum heart rate computed for his or her age (HRmax = 220), (2) the participant was unable to continue walking, and (3) the Borg scale score exceeded RPE > 17 (very difficult).

### 2.3. Instruments

**Figure 1:**
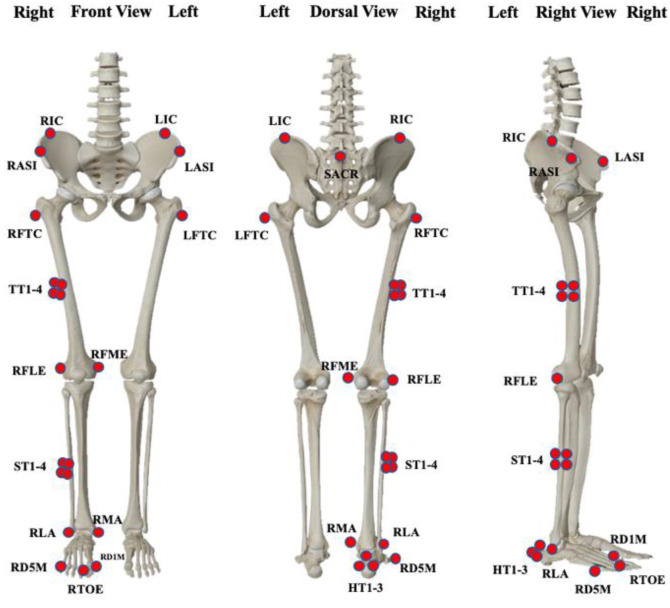
Illustration of the marker set attached to the lower extremity.

The Vicon 3D analysis system (Vicon Metrics Ltd., Oxford, UK) with eight infrared cameras was utilized to acquire kinematic data of the lower limbs at 200 Hz. Twenty-five reflex markers (9.00 mm in diameter) were used to designate the pelvis, thigh, shank, forefoot, and rearfoot segments (Figure 1) [44]. Vertical ground reaction forces were measured using AMTI force plates (AMTI, Watertown, MA, USA) at 1000 Hz. All individuals were required to wear uniform tight-fitting shorts, socks, and running shoes for the test. Data on gait kinematics and kinetics were collected from people walking at three different speeds: normal, 25% faster, and maximal. During testing, we used a velocimeter (Smart speed, Fusion Sports Inc., Burbank, CA, USA) to control the walking speed. We needed the participants’ post-fatigue gait speed to be similar to their pre-fatigue pace, and the speed error between pre-and post-fatigue testing needed to be less than 0.2 m/s [45]. Before and after the walking training, each participant had to gather three sets of gait data at different speeds.

### 2.4. Data Reduction

The kinematic data were preprocessed with Vicon Nexus software to capture the whole walking phase, process any missing marker points, and eliminate any inaccurate or duplicated marker points. The initial kinematic and kinetic data were further processed using Visual 3D software (v3; C-Motion, Inc., Germantown, MD, USA), and fourth-order Butterworth low-pass filters with cutoff frequencies of 15 and 50 Hz, respectively, were employed for smoothing [46]. Three-dimensional lower extremity joint angles were calculated concerning the proximal segment using a Cardan XYZ rotation sequence, a sequence representing flexion/extension, abduction/adduction, and axial rotation [37,47]. In the experiment, the stance phase of the gait was measured using a 10 N threshold for vertical ground response forces. Following that, joint angles and joint moments were standardized to 101 data points, each representing 1% of the stance phase.

In this study, we focused on the stiffness variations in the lower extremity joints during single-leg support (between foot flat and heel lift). The lower limb hip stiffness (k_hip_), knee stiffness (k_knee_), and ankle stiffness (k_ankle_) were calculated during the active work phase by graphing the slope of the linear regression between the bending/extension moment in the sagittal plane of each joint and the bending/extension angle during stance [48]. The least-square linear regression equation developed in MATLAB (MathWorks, Natick, MA, USA) was utilized to quantify joint stiffness. The change in joint moment M divided by the change in joint angle during stance was used to compute joint stiffness [40,49,50]:

k_joint_ = ∆M/∆θ (1)

where ΔM equals the change in joint moment, and Δθ equals the change in joint angle.

After measuring the stiffness of each joint, the determinant (r^2^) of these joints is computed to evaluate the data’s linearity; if r^2^ is more extensive than 0.80, the phase is regarded to have a moment-angle relationship that is extremely close to linear behavior [41].

### 2.5. Statistical Analysis

The Shapiro-Wilk test was done to ensure that the data distribution was normal. Mean and standard deviation was used to give descriptive statistics. A two-way ANOVA (speed × fatigue) was used, followed by a post hoc analysis when a main or interaction effect was detected. The follow-up pairwise comparison alpha level was set to 0.05 divided by the number of comparisons. A Sidak post hoc test for repeated measures analysis of variance (ANOVA) was employed (normal speed, 25% faster speed, and maximal speed). Furthermore, Pearson product-moment correlation was conducted to estimate the association between walking speed and joint stiffness. Paired t-tests were performed to determine differences in individual joint stiffness at the same walking pace before and after training. SPSS was used for all statistical analyses (version 26, SPSS Inc., Chicago, IL, USA).

## 3. Results

Participants walked at normal speed, 25% faster, and at maximum speed, most of the analyzed phases had a nearly linear moment–angle relationship (i.e., r^2^ > 0.80).

### 3.1. Hip Joint

**Figure 2:**
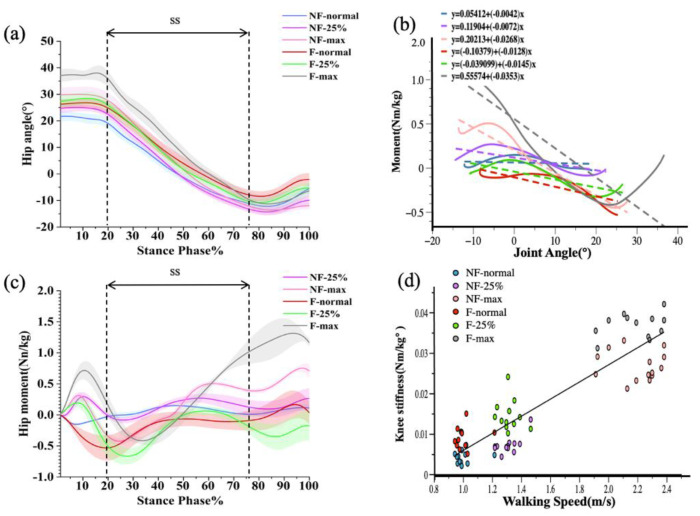
Graphical representations of the ensemble mean and standard deviation for hip angle (**a**) and hip moment (**b**). Joint-moment plots representing the three walking conditions before and after walking training (**c**), with joint stiffness calculated for the single-leg support phase (SS). Correlation plots of hip joint stiffness with walking speed before and after walking training (**d**).

In the case of hip joint stiffness, there was an interaction effect between fatigue and velocity (*p* < 0.001). As shown in Figure 2, repeated measures ANOVA showed a significant main effect on speed, with hip angle, hip moment, and hip stiffness all differing as walking speed changed during the single-leg support (SS) period (*p* < 0.001). Post hoc tests showed that increases in hip angle, moment, and stiffness were significantly different between normal speed and 25% faster speed and between normal speed and maximum speed, and between 25% faster speed, and maximum speed (*p* < 0.001). In addition, walking speed was significantly and positively correlated with hip stiffness (r = 0.81241, *p* < 0.001). Using paired t-tests, hip stiffness before and after walking was compared in the three walking conditions (normal speed, 25% faster speed, and maximum speed). As shown in Table 1, hip stiffness after walking training was significantly greater in all three walking conditions than before walking (*p* < 0.001).

### 3.2. Knee Joint

**Figure 3:**
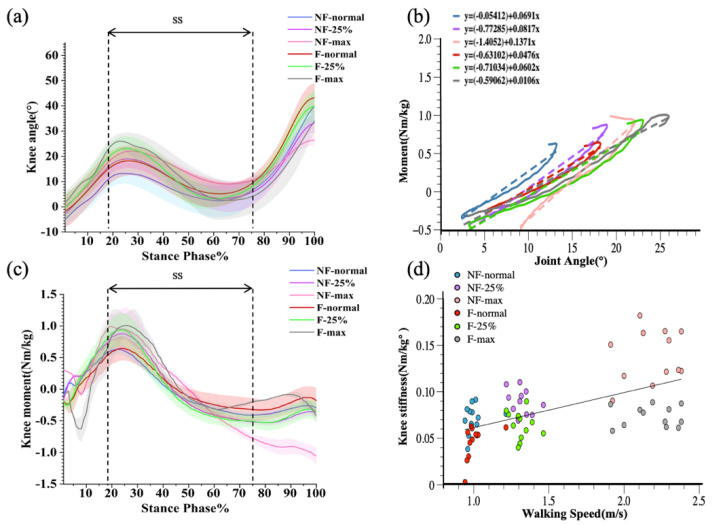
Graphical representations of the ensemble mean and standard deviation for knee angle (**a**) and knee moment (**b**). Joint-moment plots representing the three walking conditions before and after walking training (**c**), with joint stiffness calculated for the single-leg support phase (SS). Correlation plots of knee joint stiffness with walking speed before and after walking training (**d**).

In the case of knee joint stiffness, there was an interaction effect between fatigue and velocity (*p* < 0.001). As shown in Figure 3, repeated measures ANOVA showed a significant main effect on speed, with knee angle, knee moment, and knee stiffness differing as walking speed changed in the single-leg support (SS) period (*p* < 0.001). Post hoc tests showed that the increases in knee angle, moment, and stiffness were significantly different between normal speed and 25% faster speed, between normal speed and maximum speed, and between 25% faster speed and maximum speed (*p* < 0.001). In addition, walking speed was significantly and positively correlated with knee stiffness (r = 0.33217, *p* < 0.001). Paired t-tests comparing knee stiffness before and after walking in the three walking conditions (normal speed, 25% faster speed, and maximum speed) showed that knee stiffness after walking training was significantly lower than that before training for all three walking conditions, as shown in Table 1 (*p* < 0.001).

### 3.3. Ankle Joint

**Figure 4:**
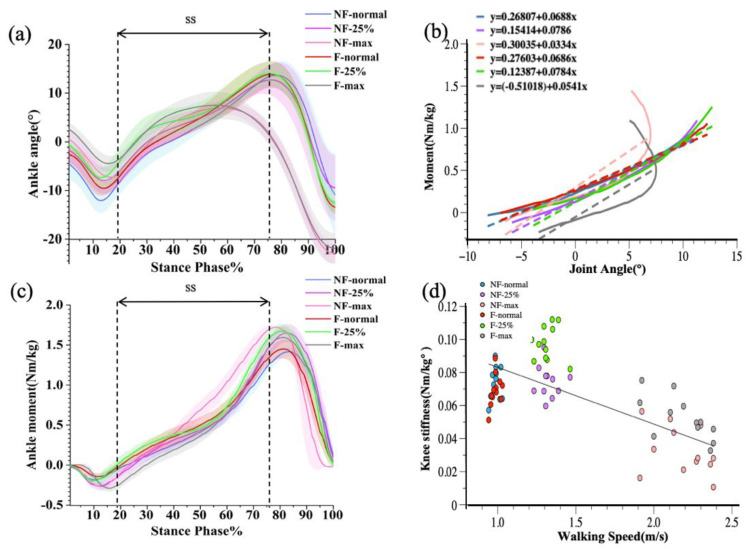
Graphical representations of the ensemble mean and standard deviation for ankle angle (**a**) and ankle moment (**b**). Joint-moment plots representing the three walking conditions before and after walking training (**c**), with joint stiffness calculated for the single-leg support phase (SS). Correlation plots of ankle joint stiffness with walking speed before and after walking training (**d**).

In the case of ankle joint stiffness, there was no interaction effect between fatigue and velocity (*p* > 0.05). As shown in Figure 4, repeated measures ANOVA showed a significant main effect on velocity (*p* < 0.001). Post hoc tests showed that increases in ankle angle, moment, and stiffness were significantly different between normal and maximal speed and between 25% faster speed and maximal speed (*p* < 0.001) but were not statistically significant between normal and 25% faster speed (*p* > 0.05). Ankle stiffness at maximal speed was significantly lower than at normal speed and at 25% faster speed. In addition, walking speed was significantly negatively correlated with knee stiffness (r = −0.827156, *p* < 0.001). Paired t-tests comparing ankle stiffness before and after walking in the three walking conditions (normal speed, 25% faster speed, and maximum speed) showed that ankle stiffness before and after training was statistically significant only at maximum speed and was significantly greater after walking training than before walking training (*p* < 0.001), while there were no obvious differences in ankle stiffness between the remaining two walking conditions (*p* > 0.05).

## 4. Discussion

The purpose of this study was to compare the stiffness of each lower extremity joint before and after walking fatigue in healthy adults walking at various speeds. By analyzing differences in lower limb joint stiffness before and after walking fatigue, this study contributes to our understanding of the adaptive pattern of lower limb biomechanics during walking. Both hip and knee stiffness had an interactive effect on walking speed and fatigue. This indicates that the proximal segment joints are more susceptible to the effects of these two walking factors.

### 4.1. The Effect of Fatigue on Stiffness

Under the same walking conditions, we found that hip stiffness was significantly higher in exhaustion than that before training (*p* < 0.001). This occurrence might be linked to such walking features as the lower load on the hip joint while walking and the stronger fatigue resistance of the hip joint supported by vast muscle groups compared to the other two joints [51,52]. As a result, following walking fatigue, even if the individual is near exhaustion in subjective assessments and heart rate data, the hip joint may benefit from muscle activation during exercise because the load is low or has not yet reached a level of fatigue, and prolonged low-intensity training has been conducted. This provides some reference value for persons whose hip and surrounding tissue have been damaged. After fatigue, the hip joint may need to carry a higher load to resist external pressures, especially during rapid walking, which may increase the demand on the hip joint. We also discovered that the stiffness of the knee joint after exhaustion was considerably lower than that before training (*p* < 0.001). Prior studies have shown that the knee joint has a high mechanical energy absorption capacity during exercise [33,51], implying that the knee joint may be capable of quickly adapting to and resisting changes in external pressures. This study found that, following repetitive flexion/extension during walking training, the knee joint’s capacity to resist external stresses may be diminished due to prolonged walking training. Because of the instability, this may increase the risk of injury in the knee joint. Prolonged exercise during walking training may cause knee joint instability. As the proximal joint, the hip actively absorbs and counteracts more mechanical changes to maintain body stability, which explains why hip stiffness is significantly greater after training than before training. When the ankle joint stiffness at the same walking speed before and after fatigue was compared, it was discovered that the stiffness at maximum speed was significantly different. The ankle joint stiffness was much higher after fatigue than that before the intervention. This is a departure from prior research on ankle stiffness during walking [53,54], possibly because the phase investigated in this study was the single-leg support period, whereas previous investigations have focused on ankle stiffness changes during force absorption. It is worth noting that the maximum walking speed in this study vastly exceeded that of prior investigations, and ankle joint stiffness did not alter appreciably at normal or 25% faster speeds, with only maximum speed producing significant changes before and after exhaustion [55]. This revealed that the lower extremity segmental pressure is modified during exercise by distributing energy absorption and production for different tasks and an adaptation mechanism to decrease tiredness. Simultaneously, in exhaustion, the ankle joint may develop a potential stress injury at maximal step speed.

As previously discussed, comparing the magnitude of stiffness of each joint before and after walking fatigue, it was confirmed that knee stiffness after walking fatigue is significantly lower than before training. In contrast, ankle stiffness at maximum walking speed produces differences before and after training and is significantly higher than that before training. Comparing the increase in hip stiffness after exhaustion may support the prior idea regarding the likelihood of energy being transferred to greater and more proximal muscle groups during exercise [51,53]. More work will be required to measure more parameters of the three joints in a state of walking fatigue to explain the interactions between the joints and the fatigue adaption patterns.

### 4.2. The Effect of Walking Speed on Stiffness

Prior studies have shown that hip joint stiffness rises with walking speed and varies dramatically over different gait cycles [40,56]. When an individual walks, the angle and moment of the hip joint fluctuate with speed, and the angle of the hip joint increases with step length in rapid walking [57]. In this study, hip stiffness increased significantly as walking speed increased, and hip stiffness was highly positively linked with speed both before and after fatigue (e.g., Figure 2d). The increase in hip stiffness during fast walking may improve power transmission [58], which matches the findings of numerous previous investigations.

Similarly, speed had a more significant effect on knee joint stiffness during walking. This finding is similar to the findings of other prior studies [55,59,60], which show that knee joint stiffness tends to increase as speed increases. The rise in speed may be followed by an increase in stride length, resulting in increased energy absorption in the knee joint to sustain athletic performance [38]. Although knee stiffness was substantially reduced after extended exercise, it increased with speed. This demonstrates that, when velocity increases after physical fatigue, the knee joint may remain actively engaged in maintaining body stability and optimizing energy expenditure during gait by increasing knee stiffness during the support phase. Fast walking may cause the center of mass to shift more during the loading period—the knee functions as the major joint for avoiding lower extremity collapse following heel impact [51,61]. As a result, after a long time of walking training, step velocity control becomes important.

At normal and 25% faster speeds, there was no significant influence of ankle joint stiffness on speed conditions before and after fatigue. The ankle joint also revealed a steady reduction in joint angle change as stride speed increased. As step speed and landing time rise, the ankle joint may adopt innate mechanical features and neural activity patterns by minimizing joint angle changes utilized to adapt to the demands of the motor task [62,63]. Ankle stiffness was independent of changes in walking speed between 1 and 1.5 m/s, which is consistent with earlier research [64,65]. In comparison to prior research on the effects of walking speed on ankle joint stiffness, this study included walking speed at maximal effort. Ankle joint stiffness was substantially different at the highest walking speed than it was at the normal and 25% faster paces, both at which ankle joint stiffness was lower. This discovery may assist in explaining the possibility of damage in patients during fast walking owing to ankle instability [48].

As walking velocity increases, the hip and knee joints are more influenced by speed, but the ankle joint is less affected. These findings might help researchers better understand gait disorders and the frequency of injuries related to fast walking. Individual joints in the lower limbs demonstrated distinct mechanical characteristics under different speeds and interventions, giving some reference foundation for trainers’ speed selection and control after exhaustion. Furthermore, knowing how lower limb joints react to variations in stiffness as walking speed rises might aid in the design of walking aids and exoskeletal devices [66,67,68,69].

### 4.3. Limitations of the Study

One disadvantage of this study is that there were no females recruited for this study, so the study results are limited to a reference population of healthy male adults rather than the whole adult population. Furthermore, various participants may have distinct exercise habits and personal peculiarities. These individual differences may cause a premature appraisal of the supervisor fatigue score of 17 in a percentage of the walking intervention subjects, resulting in the tiredness illusion, which is very important. As a result, in the following study, we shall not limit our investigation to a kinematic and kinetic standpoint. Furthermore, it is critical to confirm the pattern of changes in joint stiffness by evaluating variations in the co-activation of the antagonist and proactive lower limb muscles under the impact of fatigue and step speed. Walking may be one of the more user-friendly exercises for obese individuals; consequently, future studies will focus more on the exercise performance of obese individuals.

## 5. Conclusions

The study’s findings revealed that each joint performed differently with changes in speed before and after fatigue. Velocity has a significant impact on the mechanical performance characteristics of each joint. Knee and hip joints, in particular, showed a significant positive association with speed. Among the three walking conditions (normal speed, 25% faster speed, and maximum speed), the ankle joint stiffness revealed significant variations only at maximum speed, at which it was much lower than it was at the other two speeds. When joint stiffness before and after fatigue was compared under the same walking conditions, it was shown that hip stiffness was much higher than that before training, and knee stiffness was significantly lower than that before training. Walking fatigue appears to modify the mechanical characteristics of the joints, particularly at the maximum walking speed following fatigue, which significantly increases the load on the hip joint, according to the findings of this study. The knee joint’s lower stiffness revealed unstable features. Ankle joint stiffness differed before and after exhaustion only at the quickest pace and was much greater than the pre-training stiffness.

## Figures and Tables

**Table 1:** Joint stiffness at different step speeds before and after the exercise. Sample Mean (SD).

		Normal Speed (°/Nm Kg ^−1^)	25% Faster Speed (°/Nm Kg ^−1^)	Maximum Speed (°/Nm Kg ^−1^)	Speed × Training Significance	Speed Significance	Training Significance
**Hip Joint**	**Non-Fatigued**	0.0042 (0.0015)	0.0072 (0.0020)	0.0268 (0.0035)	*p* < 0.001	*p* < 0.001	*p* < 0.001
	**Fatigued**	0.0128 (0.0035)	0.0145 (0.0035)	0.0353 (0.0033)		*p* < 0.001	
**Knee Joint**	**Non-Fatigued**	0.0691 (0.0146)	0.0817 (0.0133)	0.1371 (0.0303)	*p* < 0.001	*p* < 0.001	*p* < 0.001
	**Fatigued**	0.0476 (0.0153)	0. 0602 (0.0143)	0.0746 (0.0106)		*p* < 0.001	
**Ankle Joint**	**Non-Fatigued**	0.0688 (0.0112)	0.0786 (0.0112)	0.0334 (0.0151)	*p* > 0.05	*p* > 0.05	*p* < 0.001 Only at maximum speed
	**Fatigued**	0.0686 (0.0117)	0.0784 (0.0141)	0.0541 (0.0208)		*p* > 0.05	

## Data Availability

The data that support the findings of this study are available on reasonable request from the corresponding author. The data are not publicly available due to privacy or ethical restrictions.
